# Effects of proline substitution/inclusion on the nanostructure of a self-assembling β-sheet-forming peptide†

**DOI:** 10.1039/d4ra07065h

**Published:** 2024-11-27

**Authors:** Jacek K. Wychowaniec, Martin Šrejber, Niting Zeng, Andrew M. Smith, Aline F. Miller, Michal Otyepka, Alberto Saiani

**Affiliations:** a Department of Materials, Manchester Institute of Biotechnology, School of Natural Sciences, Faculty of Science and Engineering, The University of Manchester UK; b AO Research Institute Davos Clavadelerstrasse 8 Davos 7270 Switzerland jacek.wychowaniec@aofoundation.org; c Regional Centre of Advanced Technologies and Materials, Czech Advanced Technology and Research Institute (CATRIN), Palacký University Olomouc 779 00 Olomouc Czech Republic; d Department of Chemical Engineering, Manchester Institute of Biotechnology, School of Engineering, Faculty of Science and Engineering, The University of Manchester UK; e IT4Innovations, VSB-Technical University of Ostrava 708 00 Ostrava-Poruba Czech Republic; f Division of Pharmacy and Optometry, Manchester Institute of Biotechnology, School of Health Sciences, Faculty of Biology, Medicine and Health, The University of Manchester UK a.saiani@manchester.ac.uk

## Abstract

Self-assembling peptides remain persistently interesting objects for building nanostructures and further assemble into macroscopic structures, *e.g.* hydrogels, at sufficiently high concentrations. The modulation of self-assembling β-sheet-forming peptide sequences, with a selection from the full library of amino acids, offers unique possibility for rational tuning of the resulting nanostructured morphology and topology of the formed hydrogel networks. In the present work, we explored how a known β-sheet-disassembling amino acid, proline (P), affects the self-assembly and gelation properties of amphipathic peptides. For this purpose, we modified the backbone of a known β-sheet-forming peptide, FEFKFEFK (F8, F = phenylalanine, E = glutamic acid, and K = lysine), with P to form three sequences: FEFKPEFK (FP), FEFKPEFKF (KPE) and FEFEPKFKF (EPK). The replacement of F by P in the hydrophobic face resulted in the loss of the extended β-sheet conformation of the FP peptide and no gelation at concentration as high as 100 mg mL^−1^, compared to typical 5 mg mL^−1^ concentration corresponding to F8. However, by retaining four hydrophobic phenylalanine amino acids in the sequences, hydrogels containing a partial β-sheet structure were still formed at 30 mg mL^−1^ for KPE (pH 4–10) and EPK (pH 2–5). TEM, AFM, small-angle X-ray scattering (SAXS) and wide-angle X-ray scattering (WAXS) revealed that KPE and EPK peptides self-assemble into nanoribbons and twisted nanofibers, respectively. Molecular dynamics confirmed that the single amino acid replacement of F by P prevented the assembly of the FP peptide with respect to the stable β-sheet-forming F8 variant. Moreover, additional prolongation by F in the KPE variant and shuffling of the polar amino acid sequence in the EPK peptide supported aggregation capabilities of both variants in forming distinct shapes of individual aggregates. Although the overall number of amino acids is the same in both KPE and EPK, their shifted charge density (*i.e.*, the chemical environment in which ionic groups reside) drives self-assembly into distinct nanostructures. The investigated structural changes can contribute to new material designs for biomedical applications and provide better understanding in the area of protein folding.

## Abstract 2

Self-assembling peptides remain persistently interesting objects for constructing defined nanostructures. By strategically incorporating proline, we can precisely control the resulting structures, ranging from flat nanoribbons to twisted nanofibers.

## Graphical abstract

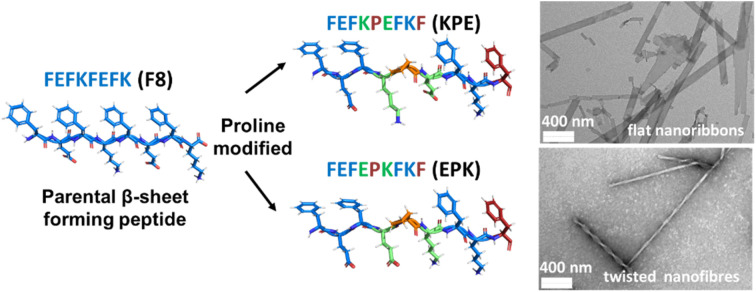

## 1. Introduction

**Fig. 1:**
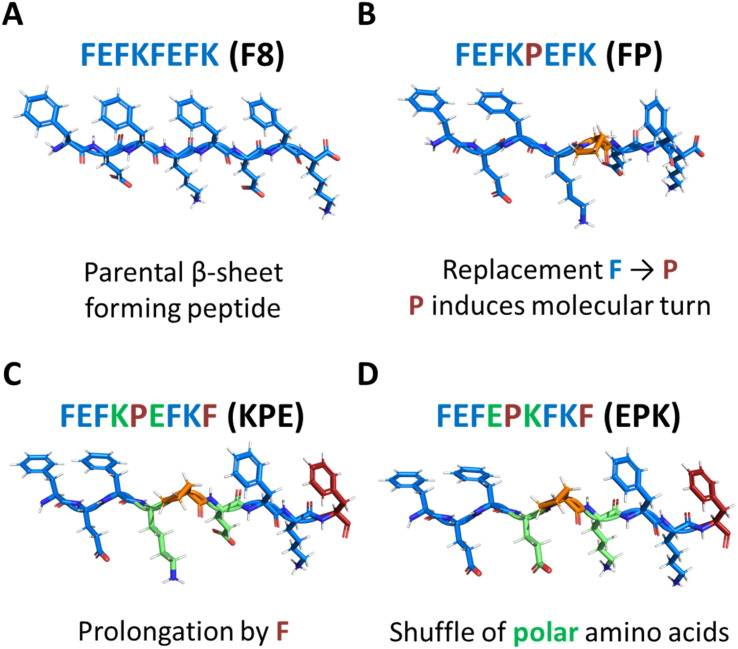
Structural models of peptides used in this study. (A) Parent β-sheet-forming peptide FEFKFEFK (F8) (F: phenylalanine, E: glutamic acid, K: lysine), and the scheme of formed nanofibres (side and top views). Chemical modification of the parent F8 peptide structure with proline (P) into 3 distinct peptides: (i) FEFKPEFK (B); (ii) FEFKPEFKF (KPE; (C)) and FEFEPKFKF (EPK; (D)).

The modulation of peptide and protein folding through careful amino acid manipulation leads to a plethora of multifunctional nanoscale material candidates for cell therapy, drug delivery and biosensors.^1–4^ In particular, over the past two decades, self-assembly of peptides into nanostructures that form hydrated networks (hydrogels) has been a widely adopted strategy for designing extracellular matrix (ECM)-mimicking scaffolds for replacing and regenerating tissues.^3,5,6^ One particular peptide design that has shown significant potential for assembly into nanofibers and formation of fully defined hydrogels for biomedical applications is based on Zhang and co-workers’ original alternated hydrophobic and hydrophilic amino acids sequences.^7^ A significant number of studies have focused on this particular design in the past two decades, and clear design rules for the formulation of hydrogels have emerged.^7–11^ Under the right conditions, well-defined short peptides (4–20 amino acid long) self-assemble into antiparallel β-sheets, in which all hydrophobic residue side groups are located on one face of the sheet, whereas all hydrophilic residue side groups are located on the opposite face. Consequently, two β-sheets further assemble to bury their hydrophobic faces and form elemental β-sheet fibres, as is the case for the typical octa-peptide FEFKFEFK (F8) (F: phenylalanine, K: lysine; and E: glutamic acid) (Fig. 1A).^12^

This peptide has been the subject of numerous studies by our group and others since its original design by Caplan *et al.*^13^ The fibres formed by this peptide and its versions with replaced amino acids have rectangular cross sections with widths of ∼3–4 nm and thicknesses of ∼1–2 nm.^12,14,15^ We have recently shown that the physiochemical properties of F8 fibres, particularly of the fibre core, surface and edges, play key roles in controlling the degree of fibre lateral association/aggregation occurring during hydrogel formation and thus play key roles in controlling the bulk properties of the formulated material.^12,15–18^ Furthermore, the effects of the position of glutamic acid and lysine have already been unravelled in the previous study, where sequence FEFEFKFK was compared to FEFKFEFK.^15^

Proline (P) is an amino acid that has been shown to be a β-sheet fibre disrupter (Fig. 1B).^19–21^ However, recent modifications using proline have been shown to: (i) favour self-assembly into twisted superhelical nanofibers,^22^ (ii) self-assemble into structures with enhanced anticancer therapeutic efficacy,^23^ or (iii) to induce the formation of lyotropic liquid crystal peptide structures.^24^ Although we previously elucidated the effect of modification of the β-sheet hydrophobic face by the introduction of bromomaleimide,^16^ the effects of introduction of proline turn in the core of the hydrophobic face have not yet been described for this class of self-assembling peptides. In particular, proline is known to induce molecular turns if correctly placed in the peptide sequence.^25^ Understanding how its position and introduction affects the structure–assembly rules still remains a challenge. In order to explore the self-assembly mechanism, we modified the original parent sequence FEFKFEFK (F8) (Fig. 1A) by replacing phenylalanine at position 5 with proline: FEFKPEFK (FP) (Fig. 1B). We further designed a new sequence in which the edge of the FP fibre was again covered with the F residue: FEFKPEFKF (KPE) (Fig. 1C) so that alternations of aromatic and polar amino acids on the two sides of proline were retained, similarly to the original F8.^15^ Finally, we shuffled polar amino acids in that sequence to shadow the previously described FEFEFKFK analogues,^15^ putting all polar glutamic acids on one side of the proline turn and lysine residues on the other: FEFEPKFKF (EPK) (Fig. 1D). Provided the importance of the hydrophobic core on the stabilization and structure of nanofibers,^12,16,17^ we reasoned that having in total four phenylalanine residues in sequences KPE and EPK with polar amino acids introduced in an alternating way will be crucial for the self-assembly into well-defined nanostructures.

To understand the effect of modifications in the fibre hydrophobic face and introduction of turn on the self-assembly of these peptides, we first performed isothermal titration calorimetry (ITC). Subsequently, the self-assembly and nanostructures of the peptide solutions at the selected pH values were investigated using a range of techniques including Fourier-transform infrared (FTIR) spectroscopy, atomic force microscopy (AFM), transmission electron microscopy (TEM), small-angle X-ray scattering (SAXS) and wide-angle X-ray scattering (WAXS). Finally, to investigate the propensity of individual peptide variants to assemble into distinct nanostructures, molecular dynamics (MD) simulation at atomistic resolutions was utilized.

## 2. Materials and methods

### 2.1. Materials

The peptides used in this work were purchased as HCl salts (>90% purity, as confirmed by the quality control HPLC measurement from the provider) from Biomatik LLC (Wilmington, Delaware, USA) and used as received. All other chemicals were purchased from Sigma-Aldrich and used as received.

### 2.2. Hydrogel preparation

Hydrogels and solutions were prepared by adding distilled water into the peptide powder to achieve concentrations from 0.1 mg mL^−1^ to 100 mg mL^−1^. After vortexing for at least 10 seconds, alternative sonication (1 h) or storing in the refrigerator overnight was applied to obtain homogeneous samples. The samples were then used as formed, or adjusted to the desired pH through gradual titration (5 μL steps) with sodium hydroxide (NaOH, 0.05 M/0.1 M/0.5 M). The pH was measured thrice using an Orion 3-Star Benchtop pH Meter (Thermo Scientific, Waltham, Massachusetts, USA).

### 2.3. Phase diagrams

The samples without pH adjustment were used as prepared at various concentrations. Samples (FP: 10 mg mL^−1^, 20 mg mL^−1^, 40 mg mL^−1^ and 100 mg mL^−1^; KEP and EPK: 5 mg mL^−1^ 10 mg mL^−1^, 20 mg mL^−1^, 30 mg mL^−1^ and 50 mg mL^−1^) were held in small test tubes with a final volume of 0.5 mL. The pH was measured at every titration point, and the gelation behaviour was recorded after each titration, with samples classified as liquid if they flowed freely upon inversion of the vials and as a gel if they did not.

### 2.4. Theoretical charge calculations and pH titration

The theoretical net charge of different peptide sequences at each pH value was calculated by the following equation:1
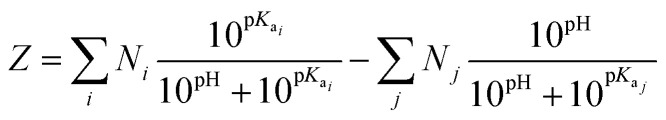
where N_*i*/*j*_ are the numbers of functional groups present on the peptide, and p*K*_a_*i*/*j*__ the p*K*_a_ are the basic (*i* – p*K*_a_ > 7) and acidic (*j* – p*K*_a_ < 7) values, respectively.^26^ Hydrophilic amino acids present on the peptides (Fig. 1) contain ionic groups that can be deprotonated, in particular carboxylic acid (COOH/COO^−^) at the C-terminus (theoretical p*K*_a_ 2.18) and on the glutamic acid side chains (theoretical p*K*_a_ 4.25), and amine (NH_3_^+^/NH_2_) at the N-terminus (theoretical p*K*_a_ 9.13 and 8.95 on the F and K side, respectively) and on the lysine side chains (theoretical p*K*_a_ 10.53).^27^ The samples without pH adjustment were used as prepared and diluted as 0.1 mg mL^−1^, 0.5 mg mL^−1^ and 1 mg mL^−1^ in final volumes of 500 μL. Different concentrations of NaOH solution were prepared (0.0025 M, 0.0125 M, 0.025 M) and titrated into the samples with different concentrations accordingly (0.1 mg mL^−1^ peptide with 0.0025 M NaOH, 0.5 mg mL^−1^ peptide with 0.0125 M NaOH and 1 mg mL^−1^ peptide with 0.025 M NaOH). During each titration, the titrant at a volume of 5 μL per drop was added and then vortexed for at least 10 seconds before the pH was measured and recorded. The titration process was repeated until a pH value of 5.0 ± 0.1 was achieved. The pH titration diagrams were obtained by plotting the peptide concentrations as a function of pH. The samples were considered thoroughly mixed, and no obvious precipitation or gelation was noticed between the pH range we tested.

### 2.5. Isothermal titration calorimetry (ITC)

The enthalpy transfer during the pH titration was measured by a heat calorimeter along with controlled pH titration by the addition of NaOH into the peptide solution. Two sample groups were measured at the same time, one was the peptide sample and the other was pure deionized water (a reference sample). Prior to the test, the samples were used as prepared (as dissolved), without any pH adjustment. Peptides were firstly diluted into 0.1 mg mL^−1^, 0.5 mg mL^−1^ and 1 mg mL^−1^, and then a final volume of 500 μL was transferred to the cell well in the testing tray of ITC. Different concentrations of NaOH were prepared (NaOH 0.0025 M, 0.0125 M, 0.025 M) and put in the wells for pipetting a volume of 200 μL in order to be titrated with the samples accordingly. Deionized water was added to the pre-rinsed wells (200 μL). The tray temperature was kept at 25 °C. One site analysis was conducted in the process. Each injection was 1.6 μL, with a total of 23 injections, and a 0.5 μL volume started the diffusion across the syringe. The energy shift diagrams were achieved by combining the pH titration diagram with the energy change diagram upon the addition of NaOH. The molar ratio of NaOH-to-peptide was calculated and the energy shift curves (kcal mol^−1^) were shown as a function of this ratio.

### 2.6. Attenuated total reflectance Fourier transform infrared (ATR-FTIR) spectroscopy

The spectra were obtained using attenuated total reflection (ATR)-Fourier transform infrared (FTIR) spectroscopy. Peptide samples (at 40 mg mL^−1^, pH adjusted to ∼4) were used as prepared and spread on the clean surface of the crystal without further adjustment after running the diagnostic test. The spectrometer (ALPHA, Bruker, Coventry, UK) was configured with a multibounce ATR plate. After the sample was loaded and the pressure arm adjusted, the sample was measured and a typical spectral peak picking was run. The resulting transmittance spectra were obtained between 1475 and 1715 cm^−1^ with a resolution of 4 cm^−1^ over 128 scans. HPLC-grade bottled water was applied as the background, and the later was subtracted from the sample spectra using the OPUS software, which was provided originally with the instrument.

### 2.7. Transmission electron microscopy (TEM)

Peptide samples were first prepared at a concentration of 20 mg mL^−1^ and then diluted with HPLC-grade water to 1 mg mL^−1^. The samples were vortexed until homogenous and then applied on grids ready for imaging. A 10 μL drop of the sample was adsorbed onto the carbon-coated copper with 400-mesh grid (sourced from Electron Microscopy Sciences) for 1 minute. A lint-free pad was then used to drain off the excess liquid. The grid was put on a 10 μL droplet of H_2_O for 10 seconds prior to the drainage of the excess liquid. Next, the grid was moved to a 10 μL droplet containing 1% uranyl acetate solution for about 30 seconds. Another 10 seconds were allowed for the drainage of the excess liquid. The grid was then stored and left until completely dry. Imaging data was taken on an FEI Tecnai12 100 kV BioTwin transmission electron microscope.

### 2.8. Atomic force microscopy (AFM)

Peptide samples were diluted from hydrogels using H_2_O to a range of concentrations (0.025 mg mL^−1^ to 0.5 mg mL^−1^). 10 μL of each dilution was dropped onto freshly cleaved mica. After 2 min, excess solution was removed, and the surface was washed once with 1 mL of HPLC-grade H_2_O. Excess water was then removed once again by wicking using Whatman No. 1 filter paper. The samples were allowed to air-dry for one night prior to imaging. Areas of interest were imaged by the scan-assist mode in air using a Bruker Multimode 8 atomic force microscope with a Nanoscope V controller operated using Nanoscope v8.15 software. Imaging was performed using ScanAsyst Air tips. These silicon nitride probes with Al coating have a nominal radius of curvature of about 2–5 nm and a nominal spring constant of 0.4 N m^−1^ (Bruker AXS S.A.S, France). Height images were captured at a scan rate of 0.977 Hz and relative humidity of <40%. The instrument was periodically calibrated using a grating with 180 nm deep, 10 mm^2^ depressions. Data were second-order flattened using the Nanoscope Analysis (v1.4) software prior to image export.

### 2.9. Small angle X-ray scattering (SAXS) and wide-angle X-ray scattering (WAXS)

SAXS/WAXS experiments were performed on beamline I22 at the Diamond Light Source (DLS) facility in Didcot, UK. The energy of the beam was 12.4 keV corresponding to the X-ray wavelength of 0.1 nm. Quartz capillaries (1.5 mm outer diameter, 0.01 mm wall thickness) were supplied from the Capillary Tube Supplies Ltd. Samples were gently introduced into the capillaries *via* a syringe. Acquisition time was 1 second and the area pixel array detector used to collect SAXS data was Pilatus P3-2M (from Dectris, Switzerland), and WAXS data was collected on Pilatus P3-2M-L (Dectris, Switzerland). The distance between the samples and the detector was fixed to 2.211 m, resulting in a momentum transfer vector range of 0.086 (nm^−1^) < *q* < 7.76 (nm^−1^) with *q* = (4π/*λ*)sin(*θ*/2), where *θ* is the scattering angle and *λ* is the wavelength of incident photons. WAXS was detected in the range from 5.72 to 60.23 nm. Calibration of the momentum transfer was performed using silver behenate powder. H_2_O in a capillary was used as the background and subtracted from all measurements, whilst the subtraction mask was created using glassy carbon. Data were reduced using the processing tools in the DawnDiamond software suite. The 2D scattering photon patterns were integrated using the azimuthal integration tool to obtain 1D scattering patterns. Fitting of elliptical cylinders was done using SasView 5.0.6 software (http://www.sasview.org/).

### 2.10. Molecular dynamics simulations (MD)

Model systems of all peptide variants (F8, FP, KPE and EPK) were studied by means of molecular dynamics simulations. Initial structures of the peptides were modelled by PyMOL (ver. 2.2.3.) software.^28^ In case of pre-assembled structures (F8 and FP variants), models were initially built as antiparallel β-sheets using the template structure of the amyloid-forming peptide (PDB ID code 3OW9 (ref. 29)). The models were built as single ladders comprising of 6 peptides and as double ladders (2 × 6 peptides) when pre-assembled along the fiber axis with the hydrophobic core packed within the structure and the hydrophilic surface exposed to water environment (Fig. S1†). The FP variant model was created by single amino acid replacement of phenylalanine by proline on parent F8 peptide (Fig. 1B and S2†). In the case of KPE and EPK variants, self-assembly conditions were created by random initial seeding of 50 individual peptides within a simulation box of 10 × 10 × 10 nm. The protonation states of all the peptides were set so that the C- and N-termini and charged polar amino acids (K, E) were in their charged forms. All molecular dynamics simulations were performed using GROMACS 5.0 simulation package.^30^ Peptide parameters were described using Amber ff99SB force field^31^ and solvated using TIP3P water model.^32^ Counter-ions were added to maintain the electroneutrality of the systems. Prior to each production run, all systems were subjected to energy minimization and subsequent thermalization was performed. Systems were heated from an initial temperature of 10 K to the desired temperature of 300 K for 10 ns under NpT conditions with the Berendsen barostat set to 1 bar.^33^ The production time was set for a minimum of 100 ns up to 500 ns under the *NVT* ensemble. The temperature was kept constant at 300 K using the v-rescale thermostat.^34^ The short-range Lennard-Jones interactions were treated with the cut-off scheme. Long-range electrostatics were calculated with the Particle Mesh Ewald (PME) summation scheme.^35^ Cut-off distances for non-bonded interactions were set to 1 nm. All bonds involving hydrogen atoms were constrained using the LINCS algorithm.^36^ All simulations had an integration time step set at 2 fs using a leap-frog algorithm. Periodic boundary conditions were set in all the directions. Table S1† shows the full list of the performed simulations.

## 3. Results and discussion

### 3.1. Self-assembly and gelation behaviour

**Fig. 2:**
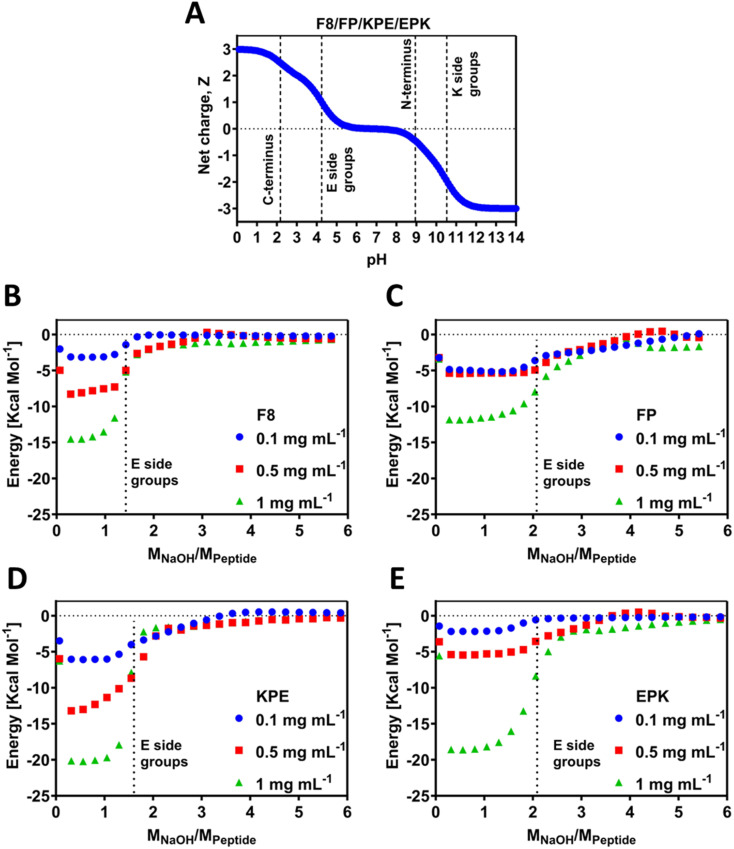
(A) Theoretical charge carried by each peptide *vs.* pH (dotted lines indicate the theoretical p*K*_a_ of the different ionic groups present on the peptides). Energy change per mole of the peptide as a function of the molar ratio of the added NaOH to the peptide for (B) FEFKFEFK (F8), (C) FEFKPEFK(FP), (D) FEFKPEFKF(KPE) and (E) FEFEPKFKF (EPK). Vertical dotted lines indicate deprotonation transition regions of the glutamic acid ionic group.

Electrostatic interactions were shown to play a key role in the self-assembly and gelation properties of this family of β-sheet-forming peptides.^13,17,37^ Previously, we have shown that titrations can shift apparent p*K*_a_ values of the amino acids due to the occurrence of self-assembly and chemical environment.^12,38^ The theoretical charge carrier by all the peptides was calculated following eqn (1) and is presented in Fig. 2A. Since all peptides contain the same amount of polar amino acids (E/K) and C- and N-terminals, in theory, all of these carry the same theoretical charge. In order to investigate the experimental behaviour for the newly designed sequences, peptides were titrated using automated isothermal thermal calorimetry (ITC) (Fig. 2B–E). ITC has been used to investigate the self-assembly process of peptides by analysis of the thermodynamic parameters.^39^ In particular, this method can be used to find the critical aggregation concentration and propensity for self-assembly, as has been elucidated for a family of RADA_4_ peptides,^40^ amelogenin,^41^ or similar to our (FKFEFKFE) enantiomeric equimolar co-assembled peptides.^42^ For ITC, the pH titration was conducted by adding stepwise small amounts of NaOH and recording the energy change during the reaction. The energy shift diagrams were achieved by plotting the energy change per mole of the peptide (*y* axis) as a function of ratio of molar concentration of the added NaOH per mole of the peptide (*x* axis). As such, this ratio (*M*_NaOH_/*M*_pep_) provides the exact number of NaOH moles required to deprotonate any polar chemical groups present on the peptide and is an experimental indication that allows to find the p*K*_a_ values of these groups. The self-assembly process is known to be dependent on the pH, and as such, any energy gains can be associated with enthalpy increase related to the deprotonation processes. By default, this process is dependent on the concentration; hence, here we studied three low concentrations of 0.1, 0.5 and 1 mg mL^−1^ (Fig. 2B–E). All peptides used for this study were purchased as HCl salts, and as a result, when solubilised in water, the pH of the initial solutions were acidic, 2.6 ± 0.2 for all peptides, and all contained the same number of glutamic acid (E) and lysine (K) residues (2 of each). The pH values are above the theoretical p*K*_a_ value of the COOH at C-terminus end groups. It can therefore be assumed that these groups are deprotonated prior to ITC experiments, whereas all other ionic groups remain protonated. As can be seen from the ITC titration curves for F8, as soon as 1 mole of NaOH per mole of peptide is added, a noticeable energy shift (enthalpy increase) occurs due to the p*K*_a_-like transition (Fig. 2B). These transitions correspond to the deprotonation of the carboxylic acid groups on the glutamic acid side chains and occur for all the three tested concentrations. After reaching ratio of 2 moles of NaOH per peptide for 0.1 mg mL^−1^ and 2.5 moles of NaOH for 0.5 and 1 mg mL^−1^, the equilibrium state was reached. Indeed, the lower amount of NaOH observed suggests a shift of roughly 0.5 pH unit toward lower values for the p*K*_a_ of the carboxylic acid side groups in F8, in agreement with the chemical titrations recently reported by us for this peptide.^12^ The extent (intensity) of energy value in ITC may be related to the strength and propensity for self-assembly, which is driven by the enthalpic advantage for the β-sheets.^39,42^ This is indeed observed by the scaled progression of the energy from 0.1 over to 1 mg mL^−1^ for F8, a known β-sheet-forming peptide.

FP peptide requires more NaOH (2 moles) for the transitions to be observed (Fig. 2C), indicating that the chemical environment of this peptide changed compared to F8. Whilst all hydrophilic amino acids remain at the same positions, only one F was replaced by P, and thus the resulting observable difference must come from the fact that the peptides do not tend to self-assemble readily into any nanostructures but remain in the monomeric form. In that form, deprotonation can occur without being influenced by closely neighbouring peptides shielded due to self-assembly. The recorded energy shift was the lowest for this peptide compared to all the tested peptides, indicative of the lack of propensity for the self-assembly for this sequence, which is further confirmed below by molecular dynamics and gelation studies. This can further be accompanied by the fact that an increase in the concentration from 0.1 to 0.5 mg mL^−1^ did not result in a large shift of enthalpy, in line with the lack of extensive self-assembly.

Peptide KPE required a similar amount of NaOH for F8, indicating that glutamic acid deprotonation also occurs for 0.5 mole NaOH earlier than predicted theoretically, and thus suggesting that self-assembly occurs, which induces the apparent observed p*K*_a_ shifts (Fig. 2D). On the other hand, it appears that the EPK peptide requires the theoretically predicted amount of NaOH for the transition to be visible (Fig. 2E). It is worth noting here that the charge density (positions of polar amino acids of E and K) is reversed compared to KPE such that both glutamic acids are closer together, neighboured by the non-ionic F. On the hydrophilic face of the peptide, these would be close to each other and not in an alternating way as in KPE, most likely leading to the occurrence of deprotonation in accordance with the theory, even if self-assembly occurs. The cause for this difference can be punitively assigned to the macroscopically observed differences, *i.e.*, gelation as well as the nanostructures formed. 0.1 and 0.5 mg mL^−1^ EPK peptides showed similar energy profiles to each other (Fig. 2E), somewhat matching the PF case and indicating that larger peptide concentrations (1 mg mL^−1^) might be required to induce self-assembly in this case. For both F8 and KPE peptides, we show a reasonable shift upon the increase in each concentration, which clearly points out the fact that self-assembly is concentration-dependent in that range.

In peptides that self-assemble into β-sheet structures, H-bonding is key in keeping several β-strands together and allowing lateral fiber growth.^13,43^ Indeed, the peptides used in this work are able to form hydrogen bonds as a number of C=O donor sites are present on both the peptide backbone. Previously, micro-differential scanning calorimetry (μDSC) was used to evaluate the peptide-polymer interactions and H-bonding.^44^ Here, the observed isothermal energy per mole of peptide will invariably measure the associated H-bonding due to the occurrence of self-assembly, with increased energy levels observed for F8, KPE and EPK peptides compared to the FP peptide (Fig. 2B–E).

**Fig. 3:**
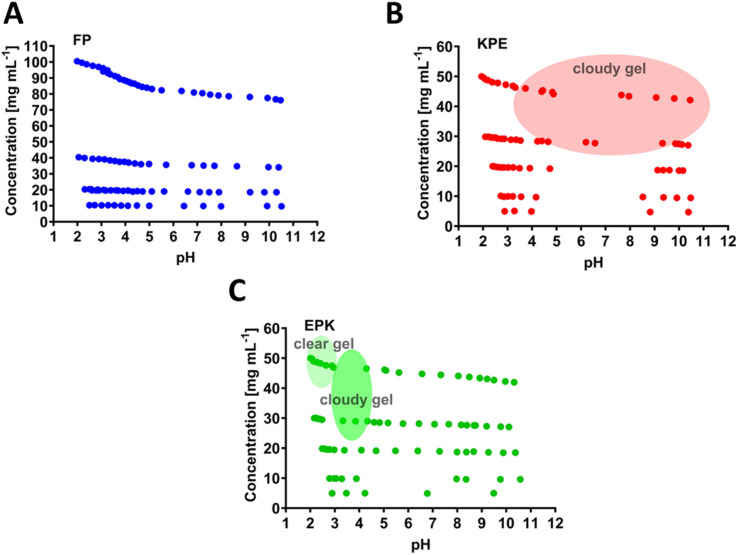
Concentration *vs.* pH phase diagrams describing the gelation region states (shaded areas) for (A) FEFKPEFK(FP), (B) FEFKPEFKF(KPE) and (C) FEFEPKFKF (EPK) peptides. Other points in the phase diagrams are noted as solution or viscous solution.

The chosen parent sequence F8 was previously shown to self-assemble readily into hydrogels at concentrations above 5 mg mL^−1^.^12^ Upon the replacement of F to P at the 5th peptide position (*i.e.*, FP sequence, Fig. 1B), we noticed a loss of gelation behaviour with no hydrogels formed even until 100 mg mL^−1^ (maximum concentration tested here, Fig. 3A). This corresponds to the measured disruption of hydrogen bonding between the two β-sheets strands and confirms the lack of self-assembly propensity of the FP peptide.

To gain a deeper insight into the structural alterations based on the proline modification of the β-sheet-forming peptide and the mechanism of peptide self-assembly, we conducted a series of molecular dynamics (MD) simulations at atomistic resolution. In terms of pre-assembled structures, plain MD simulations of the parent F8 peptide showed a great structural stability of both single and double ladder formations over the course of 500 ns (Fig. S1†). The root-mean-square deviation (RMSD) of the F8 peptide backbones did not exceed an average of 0.20 ± 0.04 nm for the single ladder form and for the 0.24 ± 0.02 nm double ladder variant for completely relaxed structures (analysis performed on the final 200 ns of production run), pointing out a great structural stability. This indicates that in both F8 forms (single and double ladder), the preservation of the original formation and structural stability originated from a favourable pre-assembled structural motif. The structure of the F8 peptide in the antiparallel β-sheet was internally supported by H-bonding interactions involving backbone atoms. An average of 7.2 ± 1.3 backbone hydrogen bonds was calculated between the internal peptide pairs (between the 3rd and 4th peptides, Fig. S3† panel A) on the single ladder form. In case of the double ladder form, an average of 7.8 ± 1.1 bonds was calculated as the overall average of backbone hydrogen bonds involving internal peptide pairs of both the chains (between the 3rd and 4th peptides and the 9th and 10th peptides, Fig. S3† panel B). Furthermore, a favourable structural arrangement with oppositely charged K and E residues with side chains aligned above each other resulted in a net of strong ionic interactions on the hydrophilic side. The hydrophobic side also supported the internal structure by the π-stacking interactions of the phenylalanine side chain moieties.

The FP variant model was also constructed in the form of a β-sheet ladder from the parent F8 peptide (Fig. S2†). However, single amino replacement of P in place of F on the parent F8 peptide introduces molecular turn, which impairs the precise alignment of vertically stacked peptides. This F to P replacement rapidly resulted in a complete loss of the pre-assembled β-sheet structure during a 100 ns simulation (Fig. S4†). The interactions were omitted into hydrophobic patches, resulting in an assembly lacking an internal structure surrounded by an aqueous environment. Even though the FP variant did not retain its pre-assembled structure, no monomerization into individual peptides was observed and the structure remained as an unordered aggregate assembly (Fig. S4†).

The KPE peptide, with added one more hydrophobic F at the end of the sequence FP (Fig. 1C), formed a cloudy gel at pH ranging from 4 to 10 (above 30 mg mL^−1^, Fig. 3B). The overall increased hydrophobicity led to larger aggregation tendency, which resulted in structures larger than visible light, where the cloudiness of the gels was observed.^12,38,45,46^ EPK peptide (Fig. 1D) formed a clear gel at a peptide concentration above 30 mg mL^−1^ but in the low pH range of 2–3 (Fig. 3C). It then turned into a cloudy gel at a pH of about 3–4 (30 mg mL^−1^ peptide), as expected, when glutamic acids undergo deprotonation transition (Fig. 2E).

### 3.2. Conformation and structure

**Fig. 4:**
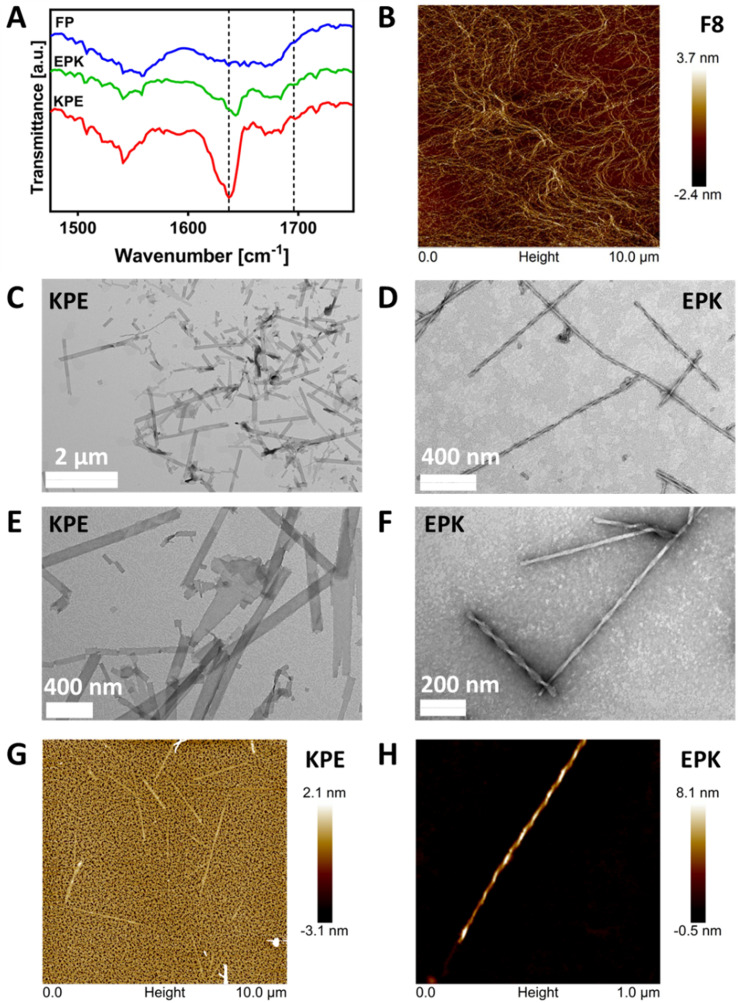
(A) FTIR-ATR normalised spectra obtained for FP, KPE and EPK hydrogels prepared at a concentration of 40 mg mL^−1^ and pH of 4.0 ± 0.5 (dotted lines indicate the position of the two bands characteristic of the adoption by peptides of the β-sheet conformations). (B) AFM image of the typical F8 network. (C–F) TEM images obtained for KPE and EPK diluted hydrogels; (G-H) AFM images obtained for KPE and EPK diluted hydrogels.

The formation of β-sheet-rich fibres was confirmed using FTIR-ATR, AFM and TEM. As can be seen from Fig. 4A in the FTIR-ATR spectra, a strong absorption band at 1636 cm^−1^ and a weaker band at 1697 cm^−1^, characteristic of the adoption by the peptides of β-sheet conformations, were observed for KPE and EPK peptides.^47,48^ On the other hand, no distinct structural feature peaks were observed for the FP peptide, confirming the lack of its tendency for self-assembly into β-sheets.

The AFM image in Fig. 4B shows the typical self-assembled peptide nanofibers of the F8 peptide forming an overall network with the thinnest fibers having diameters ranging from 3 to 5 nm, in good agreement with the formation of β-sheet-rich fibres.^12^ Unlike the typical fibres observed for FEFKFEFK or FEFEFKFK cases,^15^ the TEM images obtained and presented in Fig. 4C and E clearly show the presence of flat nanoribbons for KPE and in Fig. 4D and F well-defined twisted nanofibers for EPK, respectively. The same structures were obtained when imaged using AFM (Fig. 4G and H). The analysis of TEM micrographs reveals an average width for KPE flat nanoribbons of 72.5 ± 28.2 nm. On the other hand, EPK presents a right-handed twist, and the multi-width fibres have a similar repeating pitch in the 101–104 nm range and groove at 14–15 nm, and an average width of 26.5 ± 5.5 nm. For neither KPE nor EPK, any single fibril of ∼3–5 nm was observed, indicating that lateral stacking is strongly favoured in both cases.

**Fig. 5:**
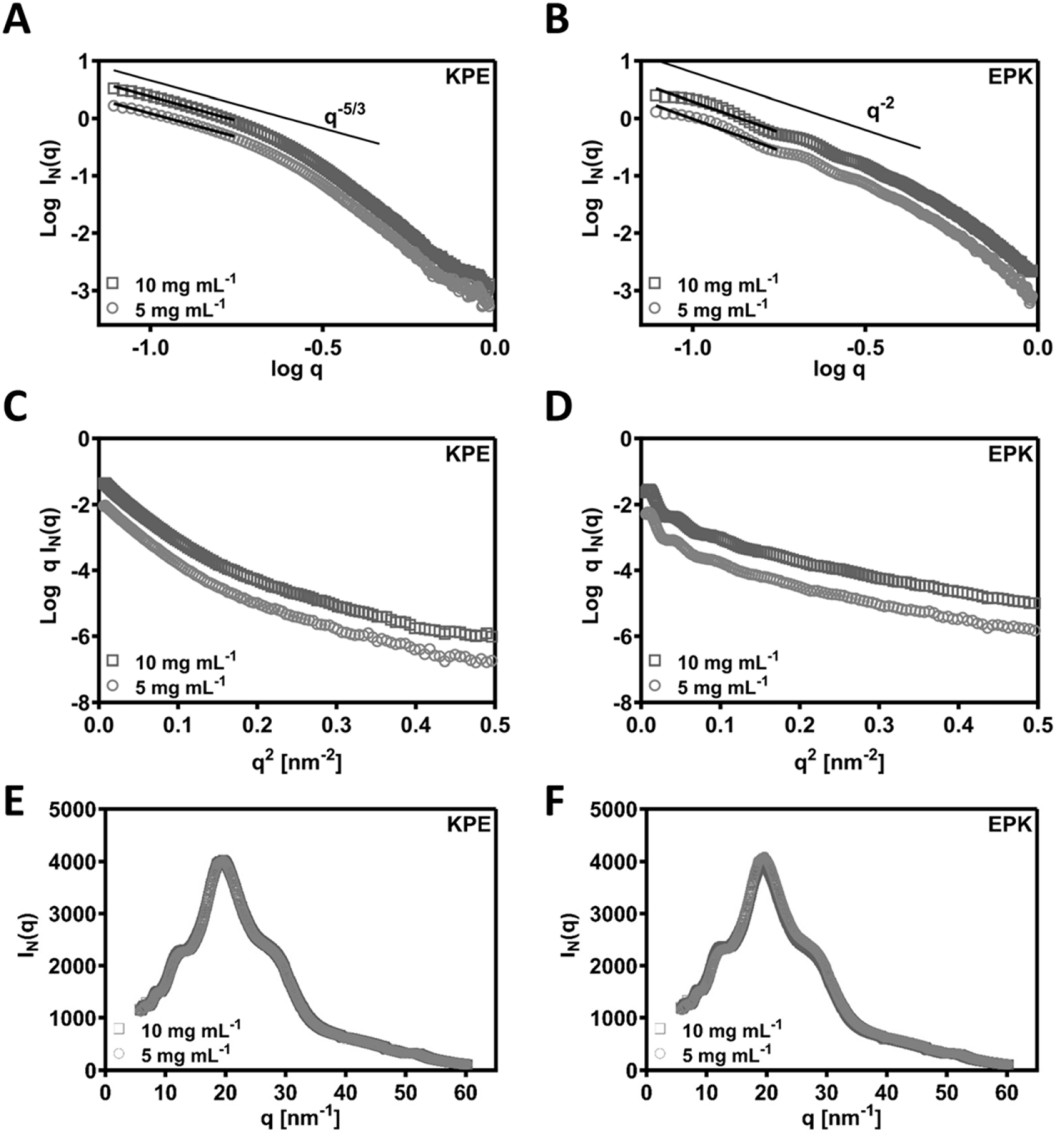
(A and B) Double logarithmic plots of the SAXS patterns obtained for the KPE and EPK samples prepared at different concentrations in *I*_*n*_(*q*) *vs. q* representation (black lines show the linear fits used to calculate the q behaviour of the two samples). SAXS patterns of (C) KPE and (D) EPK samples plotted in a ln *qI*(*q*) *vs. q*^2^ representation. Wide-angle X-ray scattering (WAXS) spectra measured as an extension to SAXS on (E) KPE and (F) EPK peptides.

SAXS was used to confirm the structural aspects. In Fig. 5A and B, the SAXS patterns obtained for KPE and EPK at two concentrations are presented as double logarithmic plots. As can be seen, *q*^−5/3^ and *q*^−2^ behaviours were observed at low *q* for KPE and EPK, respectively. These *q* regions following *q*^−2^ behaviour are often associated with flat ribbons, lamella, or disc-shaped particles.^49,50^ Both behaviours are indicative of flat ribbons, which were previously observed for arginine-based self-assembling peptides.^49,51^ In dilute regime, the SAXS data are consistent with the self-assembly of KPE into flat ribbons and EPK into twisted flatten ribbons. From the slope of the linear section of the SAXS patterns, *R*_*σ*_ of 4.05 ± 0.01 and 3.26 ± 0.06 nm was obtained for KPE and EPK, respectively. We have also fitted ellipsoidal cylinders using SasView software (Fig. S5† and Table 1) to the obtained SAXS curves and the comparison of the fittings confirmed that KPE constitutes larger structures with *R* = 4.761 ± 0.003 nm, as compared to EPK with *R* = 3.099 ± 0.003 nm. As expected from the fittings, the axis ratios and obtained lengths varied between the two structures, overall indicating that EPK forms more elongated structures with larger length as compared to KPE. The obtained characteristics go well with the obtained TEM/AFM nanostructures for these two peptides.

**Table 1:** SasView fitting of elliptical cylinders. *R* defines the ellipse minor radius, while axis ratio defines the ratio of the major radius to the minor radius. Fitting was carried out in the 0.1–7 nm^−1^ range

Sample	Conc. [mg mL^−1^]	*R* [nm]	Axis ratio	Length [nm]
KPE	5	4.761 ± 0.003	2.783 ± 0.005	733.71 ± 3.57
KPE	10	4.946 ± 0.002	2.581 ± 0.003	284.80 ± 2.34
EPK	5	3.099 ± 0.003	7.470 ± 0.030	862.39 ± 3.10
EPK	10	3.087 ± 0.002	7.650 ± 0.0224	465.40 ± 2.51

As an extension to the SAXS, we performed additional WAXS measurements and detected similar peaks for both the peptides (KPE and EPK) (by Bragg's law conversion) at 5.7 Å, 4.8 Å, 3.7 Å, 2.4 Å and 1.2 Å (Fig. 5E and F). The most prominent peak at 4.8 Å correlates well to the known 4.5 Å spacing, which is a predominant β-sheet secondary structure.^52^ The 3.7 Å spacing is ascribed to the distance between each residue along the peptide chain.^52^ The remaining peaks are indicative of a lamellar arrangement, similar to some previously obtained peptide nanostructures with helical twists.^53^ To confirm the possible arrangements of KPE and EPK peptides, we reverted back to MD simulations.

**Fig. 6:**
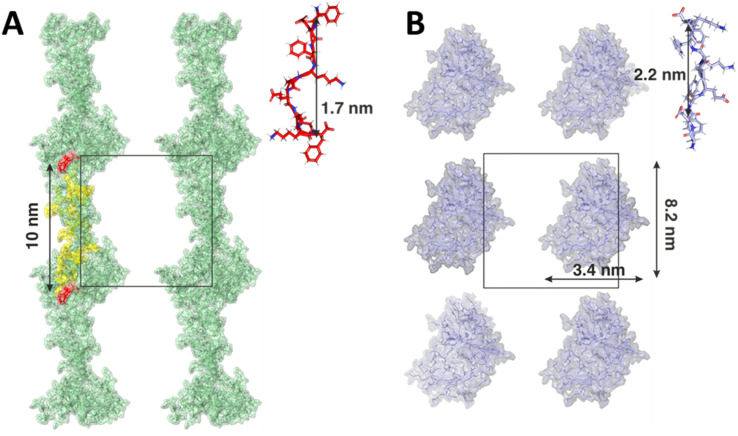
Final snapshots of (A) KPE and (B) EPK peptides. KPE residues in line with the fibre shown in yellow. The periodic box (in black) is shown for clarity. Insets show relaxed lengths of single KPE and EPK peptides.

Contrary to the F8 and FP variants, modelled as pre-assembled β-sheet ladders, we conducted simulations of KPE and EPK mimicking the self-assembly process during hydrogel preparation. In both cases, the monomers assembled rapidly, resulting in aggregates with no clearly defined secondary structure, for *e.g.*, no spontaneous aggregation into the β-sheet was observed. However, each variant assembled into different distinct shapes of aggregates. In case of the KPE variant, more fibril-like structures were observed with elongated forms connected directly across the periodic boundary conditions (Fig. 6A). The average length of a single KPE chain (measured between c-alpha carbons between the first and the last residuum on a single peptide) was 1.7 ± 0.4 nm. The distance between two mirror images of the elongated KPE form is 10 nm (corresponding to the size of the simulation box) with approximately 10 KPE molecules in line with the nanofiber. In contrast to that, the resulting shape of the EPK variant was more globular with no direct connections between the clusters across periodic boundary images (Fig. 6B). Due to the highly dynamical nature of the resulting aggregate, the width and height of the EPK form can be approximated as 3.4 nm and 8.2 nm, respectively, with the average length of one EPK chain calculated as 2.2 ± 0.1 nm. It is worth mentioning that nowadays force fields tend to bias peptides and proteins into more helical secondary structures, therefore reducing the ability to spontaneously form β-sheet-twisted fibers or nanoribbons, as counter observed in the MD simulations compared to the experiments in our case.^54^ On the one hand, by copying the 10 nm × 10 nm MD simulation box over a larger space, one can observe similarities to the experimental cases, with KPE extending over elongated and side by side nanoribbon-like assemblies, whereas EPK remains in thinner aggregates that, in principle, can grow in a twisted manner. However, we note that structural polymorphism may occur, as has been noted in β-sheet amyloid fibres research.^55^

## 4. Conclusions

In summary, we have investigated the effect of a known β-sheet breaker amino acid, proline (P), on a known β-sheet and nanofiber forming peptide FEFKFEFK through the replacement of phenylalanine in the hydrophobic face FEFKPEFK (FP) as well as the extension and shuffling of the polar amino acids to generate two new sequences: FEFKPEFKF (KPE) and FEFEPKFKF (EPK). As expected, the modification of the hydrophobic core from F to P disrupted the self-assembly and resulted in a loss of conformational stability and subsequent formation of nanostructures or hydrogelation. However, the addition of aromatic amino acids to the edge of that variant restored self-assembly into nanostructures, resulting either in flat nanoribbons or twisted nanofibers, depending on the charge distribution of the external polar amino acids. By the meticulous design of peptides and targeted placement of proline as well as the distribution of charged residues (glutamic acid and lysine), well defined nanostructures (and hydrogels) could be formed. Molecular dynamics supported the formation of well-defined aggregates, which can scale into microscopically observed nanostructures in AFM and TEM. Our work demonstrates both the destructive and non-destructive self-assembly using proline and sheds light both on the fabrication of nanostructures and supramolecular hydrogels for possible regenerative medicine applications as well as harnessing and understanding of the protein folding capabilities in a biomaterial-targeted manner.

## Data availability

All research data supporting this publication are directly available within this publication and associated ESI.†

## Author contributions

J. K. W.: conceptualization, data curation, formal analysis, investigation, methodology, validation, visualization, writing – original draft, writing – review & editing; M. Š.: data curation, investigation, formal analysis, methodology, writing – review & editing; N. Z.: data curation, investigation, methodology; A. M. S.: conceptualization, data curation, investigation, methodology, supervision; A. F. M.: resources, supervision; M. O.: resources, supervision, writing – review & editing; A. S.: conceptualization, resources, funding acquisition, project administration, writing – review & editing.

## Conflicts of interest

The authors declare no competing interests.

## Supplementary Material

RA-014-D4RA07065H-s001

## References

[cit1] Sheehan F., Sementa D., Jain A., Kumar M., Tayarani-Najjaran M., Kroiss D., Ulijn R. V. (2021). Chem. Rev..

[cit2] Sinha N. J., Langenstein M. G., Pochan D. J., Kloxin C. J., Saven J. G. (2021). Chem. Rev..

[cit3] Marchesan S., Styan K. E., Easton C. D., Waddington L., Vargiu A. V. (2015). J. Mater. Chem. B.

[cit4] Elsawy M. A., Wychowaniec J. K., Castillo Diaz L. A., Smith A. M., Miller A. F., Saiani A. (2022). Biomacromolecules.

[cit5] Mondal S., Das S., Nandi A. K. (2020). Soft Matter.

[cit6] Koutsopoulos S. (2016). J. Biomed. Mater. Res., Part A.

[cit7] Zhang S. G. (2002). Biotechnol. Adv..

[cit8] Wang Y., Zhang W., Gong C., Liu B., Li Y., Wang L., Su Z., Wei G. (2020). Soft Matter.

[cit9] Jonker A. M., Löwik D. W. P. M., van Hest J. C. M. (2012). Chem. Mater..

[cit10] De Leon Rodriguez L. M., Hemar Y., Cornish J., Brimble M. A. (2016). Chem. Soc. Rev..

[cit11] Wychowaniec J. K., Patel R., Leach J., Mathomes R., Chhabria V., Patil-Sen Y., Hidalgo-Bastida A., Forbes R. T., Hayes J. M., Elsawy M. A. (2020). Biomacromolecules.

[cit12] Wychowaniec J. K., Smith A. M., Ligorio C., Mykhaylyk O. O., Miller A. F., Saiani A. (2020). Biomacromolecules.

[cit13] Caplan M. R., Moore P. N., Zhang S. G., Kamm R. D., Lauffenburger D. A. (2000). Biomacromolecules.

[cit14] Wychowaniec J. K., Moffat J., Saiani A. (2021). J. Mech. Behav. Biomed. Mater..

[cit15] Gao J., Tang C., Smith A. M., Miller A. F., Saiani A. (2017). Biomacromolecules.

[cit16] Elsawy M. A., Smith A. M., Hodson N., Squires A., Miller A. F., Saiani A. (2016). Langmuir.

[cit17] Roberts D., Rochas C., Saiani A., Miller A. F. (2012). Langmuir.

[cit18] Dong S., Chapman S. L., Pluen A., Richardson S. M., Miller A. F., Saiani A. (2024). Biomacromolecules.

[cit19] Jacob J., Duclohier H., Cafiso D. S. (1999). Biophys. J..

[cit20] Williamson M. P. (1994). Biochem. J..

[cit21] Bełdowski P., Przybyłek M., Bełdowski D., Dedinaite A., Sionkowska A., Cysewski P., Claesson P. M. (2022). J. Mater. Chem. B.

[cit22] Ghosh M., Bera S., Schiffmann S., Shimon L. J. W., Adler-Abramovich L. (2020). ACS Nano.

[cit23] Li M., Ning Y., Chen J., Duan X., Song N., Ding D., Su X., Yu Z. (2019). Nano Lett..

[cit24] Pelin J. N. B. D., Edwards-Gayle C. J. C., Castelletto V., Aguilar A. M., Alves W. A., Seitsonen J., Ruokolainen J., Hamley I. W. (2020). ACS Appl. Mater. Interfaces.

[cit25] Nilsson I., Sääf A., Whitley P., Gafvelin G., Waller C., von Heijne G. (1998). J. Mol. Biol..

[cit26] Edwards-Gayle C. J. C. and Wychowaniec J. K., in Peptide Bionanomaterials, ed. M. A. Elsawy, Springer International Publishing, Cham, 2023, ch. 8, pp. 255–308, 10.1007/978-3-031-29360-3_8

[cit27] Nelson D. L. , Lehninger A. L. and Cox M. M., Lehninger Principles of Biochemistry, Macmillan Higher Education, Basingstoke, 7th edn, 2017

[cit28] The PyMOL Molecular Graphics System, Version 2.2.3, Schrodinger, LLC

[cit29] Colletier J.-P., Laganowsky A., Landau M., Zhao M., Soriaga Angela B., Goldschmidt L., Flot D., Cascio D., Sawaya Michael R., Eisenberg D. (2011). Proc. Natl. Acad. Sci. U. S. A..

[cit30] Van Der Spoel D., Lindahl E., Hess B., Groenhof G., Mark A. E., Berendsen H. J. C. (2005). J. Comput. Chem..

[cit31] Hornak V., Abel R., Okur A., Strockbine B., Roitberg A., Simmerling C. (2006). Proteins: Struct., Funct., Bioinf..

[cit32] Jorgensen W. L., Chandrasekhar J., Madura J. D., Impey R. W., Klein M. L. (1983). J. Chem. Phys..

[cit33] Berendsen H. J. C., Postma J. P. M., van Gunsteren W. F., DiNola A., Haak J. R. (1984). J. Chem. Phys..

[cit34] Bussi G., Donadio D., Parrinello M. (2007). J. Chem. Phys..

[cit35] Darden T., York D., Pedersen L. (1993). J. Chem. Phys..

[cit36] Hess B., Bekker H., Berendsen H. J. C., Fraaije J. G. E. M. (1997). J. Comput. Chem..

[cit37] Aggeli A., Bell M., Boden N., Carrick L. M., Strong A. E. (2003). Angew. Chem., Int. Ed..

[cit38] Wychowaniec J. , Bektas E. I., Mürner M., Sapudom J., Šrejber M., Airoldi M., Schmidt R., Vernengo A., Edwards-Gayle C., Tipay P., Otyepka M., Teo J., Eglin D. and D’Este M., Self-assembly of Tyrosine-containing Peptides into Injectable Hydrogels with Distinct Nanostructures is Key in Determining Inflammatory Response of Macrophages, ChemRxiv, 2024, preprint, 10.26434/chemrxiv-2024-bn5sm-v2

[cit39] Kabiri M., Unsworth L. D. (2014). Biomacromolecules.

[cit40] Kabiri M., Bushnak I., McDermot M. T., Unsworth L. D. (2013). Biomacromolecules.

[cit41] Lakshminarayanan R., Yoon I., Hegde B. G., Fan D., Du C., Moradian-Oldak J. (2009). Proteins: Struct., Funct., Bioinf..

[cit42] Swanekamp R. J., DiMaio J. T. M., Bowerman C. J., Nilsson B. L. (2012). J. Am. Chem. Soc..

[cit43] Caplan M. R., Schwartzfarb E. M., Zhang S. G., Kamm R. D., Lauffenburger D. A. (2002). Biomaterials.

[cit44] Maslovskis A., Tirelli N., Saiani A., Miller A. F. (2011). Soft Matter.

[cit45] Aggeli A., Bell M., Carrick L. M., Fishwick C. W. G., Harding R., Mawer P. J., Radford S. E., Strong A. E., Boden N. (2003). J. Am. Chem. Soc..

[cit46] Boothroyd S., Miller A. F., Saiani A. (2013). Faraday Discuss..

[cit47] Barth A. (2007). Biochim. Biophys. Acta, Rev. Bioenerg..

[cit48] Barth A., Zscherp C. (2002). Q. Rev. Biophys..

[cit49] Hamley I. W. (2008). Macromolecules.

[cit50] Nguyen A. K., Molley T. G., Kardia E., Ganda S., Chakraborty S., Wong S. L., Ruan J., Yee B. E., Mata J., Vijayan A., Kumar N., Tilley R. D., Waters S. A., Kilian K. A. (2023). Nat. Commun..

[cit51] Hamley I. W., Dehsorkhi A., Castelletto V. (2013). Chem. Commun..

[cit52] Pugliese R., Marchini A., Saracino G. A. A., Zuckermann R. N., Gelain F. (2018). Nano Res..

[cit53] Bairagi D., Biswas P., Basu K., Hazra S., Hermida-Merino D., Sinha D. K., Hamley I. W., Banerjee A. (2019). ACS Appl. Bio Mater..

[cit54] Best R. B., Buchete N.-V., Hummer G. (2008). Biophys. J..

[cit55] Ke P. C., Zhou R., Serpell L. C., Riek R., Knowles T. P. J., Lashuel H. A., Gazit E., Hamley I. W., Davis T. P., Fändrich M., Otzen D. E., Chapman M. R., Dobson C. M., Eisenberg D. S., Mezzenga R. (2020). Chem. Soc. Rev..

