# Supplementary material for: Effects of proline substitution/inclusion on the nanostructure of a self-assembling β-sheet-forming peptide
Source: RSC Adv. 2024 Nov 27;14(50):37419–30. doi: 10.1039/d4ra07065h (PMC11601148; doi:10.1039/d4ra07065h)
Supplement: RA-014-D4RA07065H-s001 [file RA-014-D4RA07065H-s001.pdf]

Supplementary Information (SI) for RSC Advances.
This journal is © The Royal Society of Chemistry 2024

Effects of Proline Substitution/Inclusion on the Nanostructure of Self-assembling β-Sheet Forming Peptide – Supporting Information

Jacek K. Wychowaniec[1,2,3,*], Martin Šrejber[4], Niting Zeng[1,2], Andrew M. Smith[1,2], Aline F. Miller[2,5], Michal Otyepka[4,6], Alberto Saiani[1,2,*]

[1] Department of Materials, University of Manchester, Oxford Road, Manchester M13 9PL, UK

[2] Manchester Institute of Biotechnology, The University of Manchester, Oxford Road, M13 9PL, Manchester, UK

[3] AO Research Institute Davos, Clavadelerstrasse 8, 7270, Davos, Switzerland

[4] Regional Centre of Advanced Technologies and Materials, Czech Advanced Technology and Research Institute (CATRIN), Palacký University Olomouc, 779 00 Olomouc, Czech Republic

[5] Department of Chemical Engineering and Analytical Sciences, University of Manchester, Oxford Road, Manchester M13 9PL, UK

[6] IT4Innovations, VSB-Technical University of Ostrava, 708 00 Ostrava-Poruba, Czech Republic

[*] Corresponding authors:

J.K.W. e-mail: jacek.wychowaniec@aofoundation.org

A.S. e-mail: a.saiani@manchester.ac.uk

Table S1. *Detailed information of performed molecular dynamics simulations.*

		Number of peptides	*Number of H_2O molecules*	*Simulation box size [nm]*	*Simulation length [ns]*
pre-assembled	F8 single ladder	6	9347	7.6 × 6.7 × 5.7	500
	F8 double ladder	12	11521	7.8 × 6.7 × 6.9	500
	FP single ladder	6	12628	8.3 × 7.4 × 6.4	100
self-assembly	KPE	50	30002	10 × 10 × 10	100
	EPK	50	29957	10 × 10 × 10	100

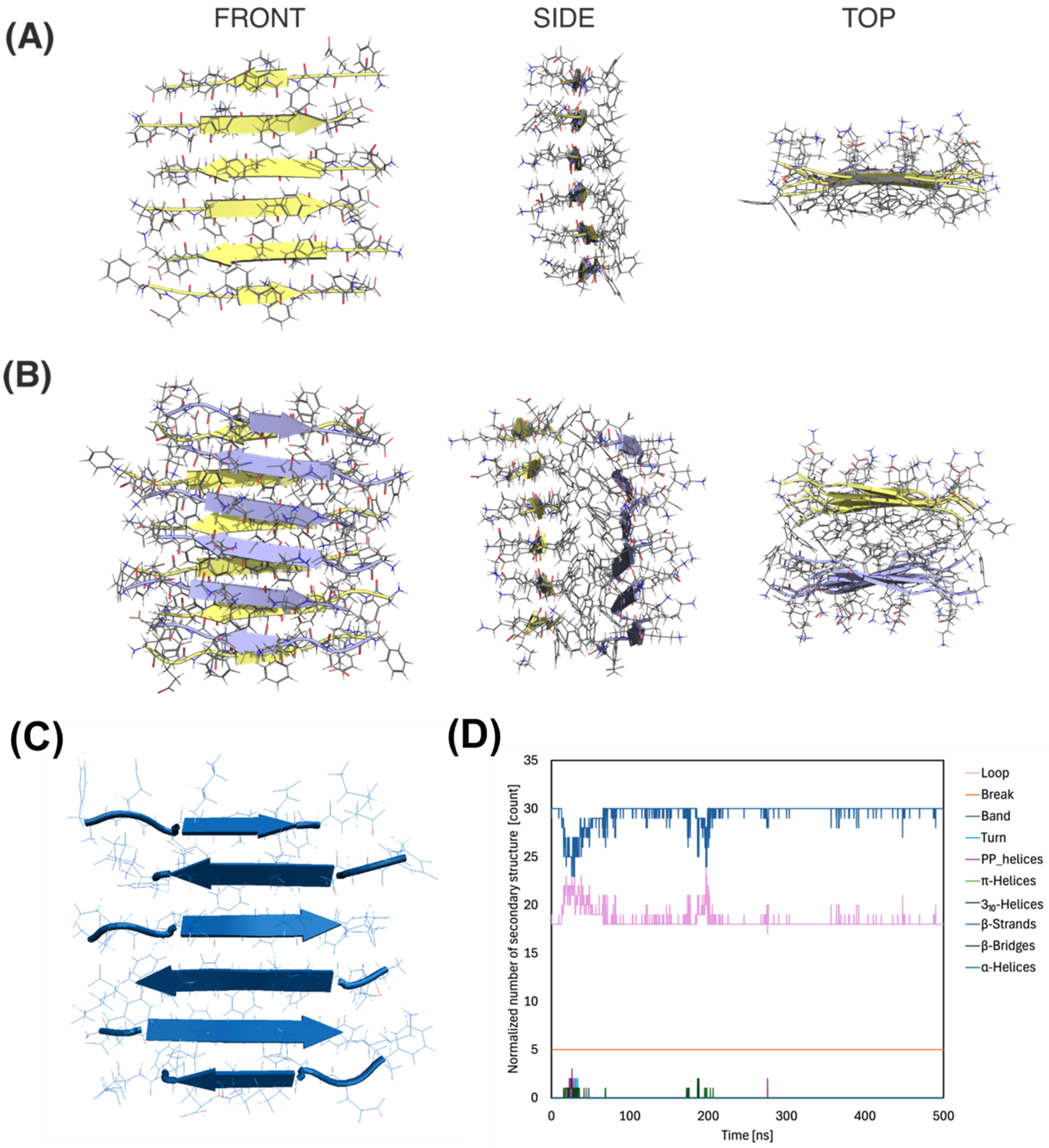

Figure S1. *Final structures of F8 peptides (front, side, and top view) pre-assembled in single ladder* ***(A)*** *and double ladder* ***(B)****.* ***(C)*** *Final snapshot of structure of F8 parental peptide sequence and* ***(D)*** *normalized secondary structural motifs as a function of time depicting a clear propensity of pre-assembled ladder conformation to form antiparallel stacking model.*

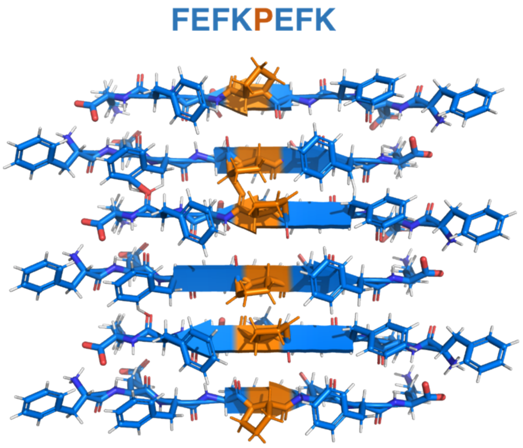

Figure S2. *The initial structure of FP variant constructed as single amino acid replacement of phenylalanine to proline in the form of β-sheet ladder from parental F8 peptide.*

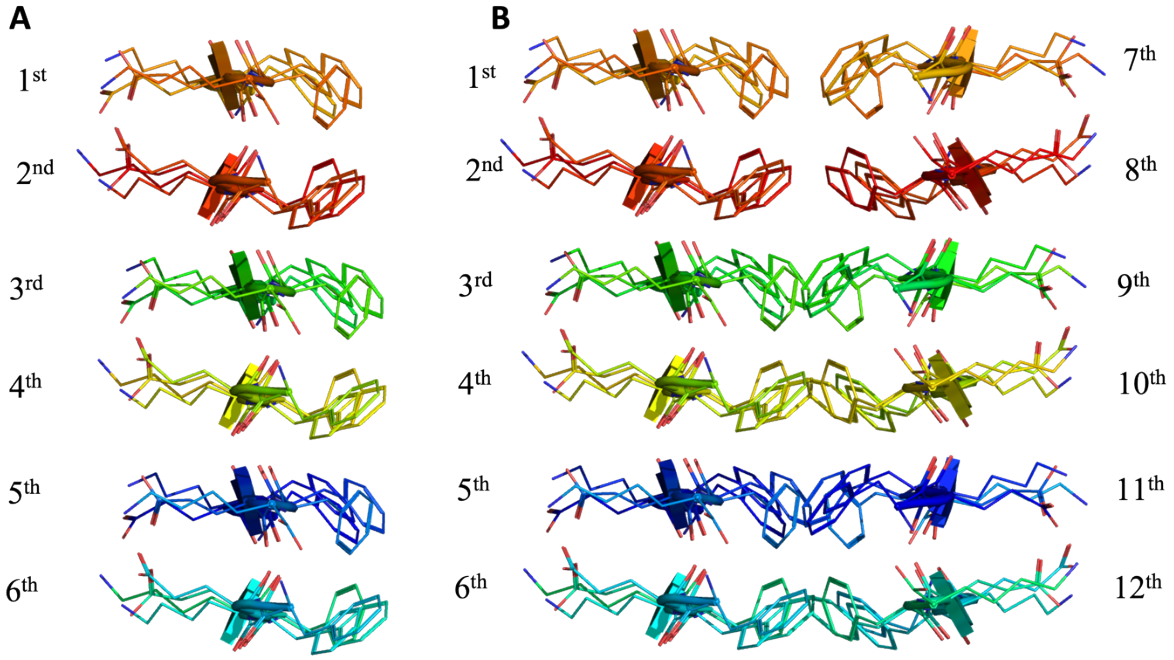

Figure S3. *Numbering of single ladder (**A**) and double ladder (**B**) forms of F8 variant.*

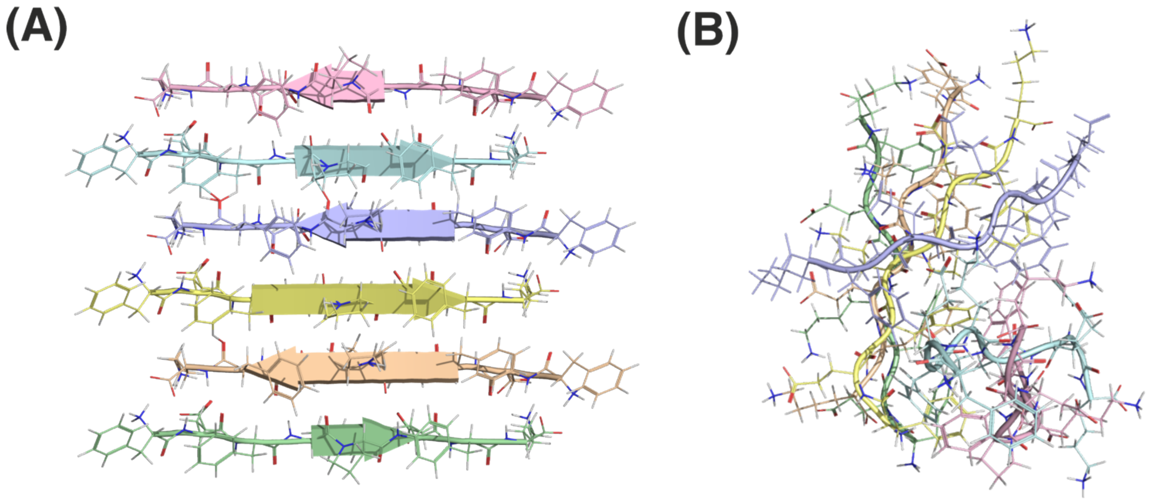

Figure S4. *The starting structure* ***(A)*** *and final snapshot* ***(B)*** *of FP variant after 100 ns.*

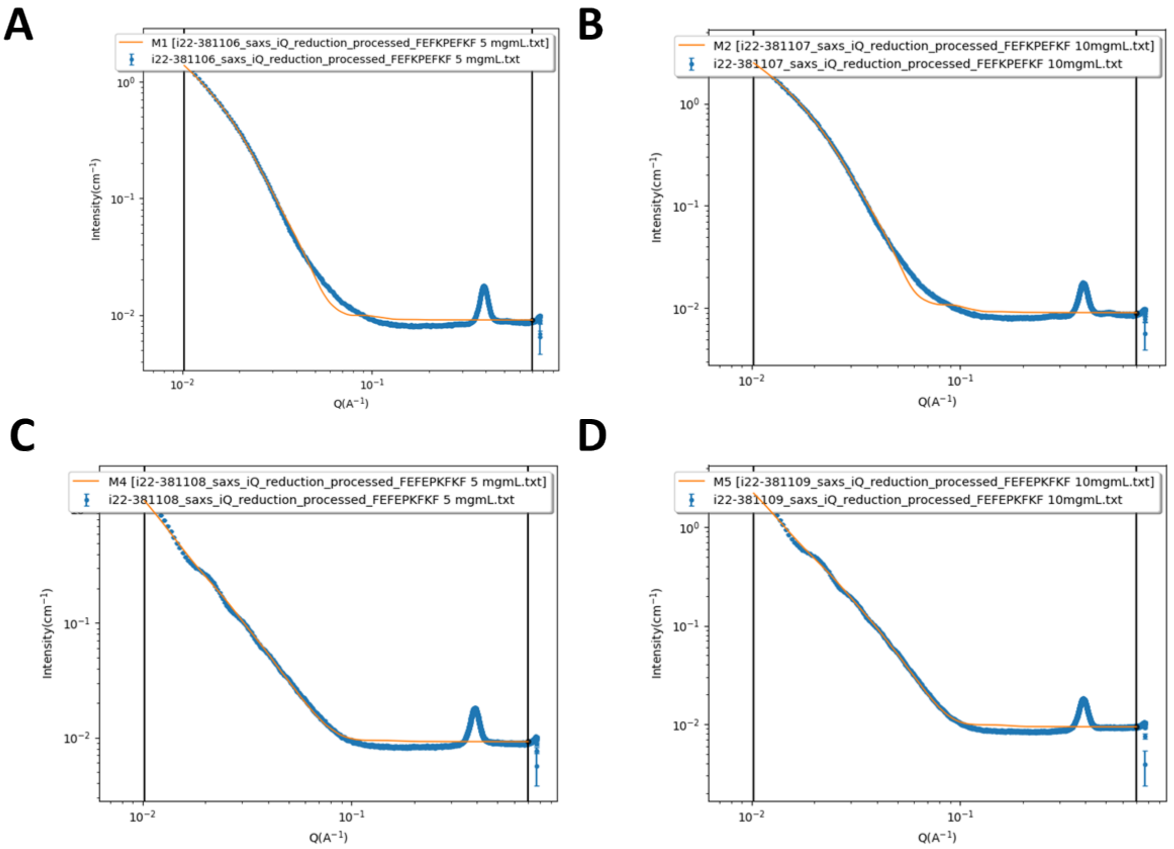

Figure S5. *SasView fittings of SAXS patterns obtained for:* ***(A)*** *5 mg mL^{-1} KPE,* ***(B)*** *10 mg mL^{-1} KPE,* ***(C)*** *5 mg mL^{-1} EPK,* ***(D)*** *10 mg mL^{-1} EPK. Orange lines show the elliptical cylinder fitting and blue lines depict original data.*
